# Follow-up of patients with myocardial ischemia without obstructive coronary lesions

**DOI:** 10.47487/apcyccv.v6i2.472

**Published:** 2025-06-27

**Authors:** Ana Liuvys Cuba Rodríguez, Lázaro Isralys Aldama Pérez, Myder Hernández Navas, Snayder José Goelkel Pérez

**Affiliations:** 1 Centro de Investigaciones Médico-Quirúrgicas (CIMEQ), La Habana, Cuba. Centro de Investigaciones Médico Quirúrgicas Centro de Investigaciones Médico-Quirúrgicas (CIMEQ) La Habana Cuba; 2 Departamento de Hemodinámica y Cardiología Intervencionista, Hospital Clínico Quirúrgico Hermanos Ameijeiras, La Habana, Cuba. Departamento de Hemodinámica y Cardiología Intervencionista Hospital Clínico Quirúrgico Hermanos Ameijeiras La Habana Cuba; 3 Instituto de Medicina Deportiva, La Habana, Cuba Instituto de Medicina Deportiva La Habana Cuba; 4 Universidad Ciencias Médicas de La Habana, Cuba Universidad de Ciencias Médicas de La Habana Universidad Ciencias Médicas de La Habana Cuba

**Keywords:** Microcirculation, Myocardial Ischemia, Coronary Angiography, Microcirculación, Isquemia Miocárdica, Angiografía Coronaria

## Abstract

**Objective.:**

The presence of coronary lesions of less than 50% in patients with myocardial ischaemia is a common diagnosis in cardiac catheterisation laboratories. The aim of this study was to determine the clinical course of patients with myocardial ischaemia in the absence of obstructive coronary lesions.

**Materials and methods.:**

A prospective analytical study was conducted in 110 patients of both sexes with documented myocardial ischaemia and coronary lesions of less than 50% on coronary angiography. The follow-up period was three years.

**Results.:**

The mean age was 64.5 ± 7.2 years, with a predominance of females (57%). The most prevalent risk factors were hypertension (58.2%) and dyslipidaemia (44.5%). In 8.3% of cases, re-hospitalisation was required, with heart failure reported as the leading cause (6.5%). Cardiovascular event-free survival during follow-up was 80%, and was higher in patients without coronary lesions. A higher incidence of cardiovascular events was associated with diabetes mellitus, a family history of ischaemic heart disease, and chronic kidney disease.

**Conclusions.:**

Patients with myocardial ischaemia in the absence of obstructive coronary lesions experienced adverse events during follow-up, particularly those with diabetes mellitus, a family history of ischaemic heart disease, and chronic kidney disease.

## RESUMEN

**Objetivo.:**

La presencia de lesiones coronarias menores al 50%, en pacientes con isquemia miocárdica, es un diagnóstico frecuente en los laboratorios de cateterismo cardíaco. El objetivo del estudio es determinar la evolución de los pacientes con isquemia miocárdica en ausencia de lesiones coronarias obstructivas.

**Materiales y métodos:**

. Se desarrolló un estudio prospectivo analítico, en 110 pacientes de ambos sexos con isquemia miocárdica documentada y lesiones coronarias menores del 50% en la angiografía coronaria, con un período de seguimiento de tres años.

**Resultados:**

. La edad promedio fue 64,5 ± 7,2, años con predominio del sexo femenino (57%). Los factores de riesgo de mayor prevalencia fueron la hipertensión arterial y la dislipidemia, con 58,2 y 44,5%, respectivamente. En el 8,3% de los casos fue necesaria una nueva hospitalización y se reportó la insuficiencia cardiaca como principal causa (6,5%). La supervivencia libre de eventos cardiovasculares adversos durante el seguimiento fue del 80%, superior en pacientes sin lesiones coronarias. La mayor incidencia de eventos cardiovasculares se relacionó con la presencia de diabetes *mellitus*, el antecedente familiar de cardiopatía isquémica y la enfermedad renal crónica.

**Conclusiones:**

. Los pacientes con isquemia miocárdica en ausencia de lesiones coronarias obstructivas mostraron eventos adversos durante el seguimiento, más frecuentes en presencia de diabetes *mellitus*, antecedentes familiares de cardiopatía isquémica y enfermedad renal crónica.

## Introduction

Ischemic heart disease is the leading cause of death and disability worldwide ^1^^,^^2^. Half or more of patients with documented myocardial ischemia do not present angiographically significant coronary lesions, defined as a stenosis diameter of less than 50% ^3^. This syndrome, globally known as INOCA (Ischemia with No Obstructive Coronary Arteries), is primarily attributed to coronary microvascular dysfunction in any of its forms, either impaired vasodilatory capacity or microcirculatory spasm ^4^.

An increase in the prevalence of INOCA has been reported over the past decade, as a result of growing recognition of the condition as a clinically important diagnosis distinct from obstructive coronary artery disease (CAD). According to data from the National Cardiovascular Data Registry of the American College of Cardiology, it is estimated that approximately 3 to 4 million individuals experience signs and symptoms of ischemia without obstructive CAD each year. However, this figure reflects only those patients referred for invasive angiography. It is important to note that the prevalence reported in the literature varies between 35% and 50%. Additionally, a higher prevalence has been observed in women (65% vs. 32% in men) ^3^^,^^5^^,^^6^.

Although previously considered to have a benign prognosis, women with INOCA are at increased risk of major adverse cardiovascular events (MACE), including a ten-fold increase in heart failure with preserved ejection fraction, stroke, and coronary microvascular dysfunction (CMD). Older age, diabetes, hypertension, and smoking are predictors of worse outcomes in patients with INOCA, including higher all-cause mortality. Moreover, patients with nonobstructive CAD affecting all three coronary arteries have an annual risk of myocardial infarction and death comparable to that of patients with single-vessel obstructive CAD ^6^.

Microvascular dysfunction has been associated with inflammatory mechanisms of various etiologies, including altered autonomic tone, disrupted ion transport across cell membranes, increased endothelin release, and estrogen deficiency ^6^^-^^8^. Its development leads to a decline in quality of life and increases the incidence of adverse cardiovascular events in affected patients ^9^.

This study aims to evaluate the clinical trajectory of patients with myocardial ischemia in the absence of angiographically significant CAD.

## Materials and methods

### Study design and population

An analytical, prospective study was conducted involving male and female patients with myocardial ischemia documented by cardiovascular stress testing. These patients exhibited coronary lesions with less than 50% stenosis based on coronary angiography findings, which were obtained in the hemodynamics laboratory at Cardiocentro CIMEQ between 2017 and 2019. Patients were selected according to predefined inclusion criteria: individuals with chronic stable angina who demonstrated high or intermediate risk based on the Duke treadmill score during cardiovascular stress testing, and whose coronary angiography revealed lesions with less than 50% stenosis. Patients with a history of coronary revascularization or acute coronary syndrome within the three months prior to inclusion were excluded.

### Cardiovascular stress testing

Cardiovascular stress testing was performed in clinically stable patients using the Bruce protocol on a treadmill. Twelve-lead electrocardiograms and blood pressure readings were recorded before, during, and after exercise. The presence of angina, dyspnea, hypotension, ventricular arrhythmias, or marked ST-segment depression (≥ 3 mm) were considered criteria for test termination. An abnormal ST-segment response during exercise was defined as horizontal or downsloping ST depression ≥ 1 mm measured 80 milliseconds after the J point, or an elevation ≥ 1 mm in leads without pathological Q waves, excluding lead aVR. Risk stratification was conducted using the Duke Treadmill Score, which estimates cardiovascular mortality based on conventional stress test results. The score is calculated as follows: (exercise time in minutes) - (5 x ST-segment deviation in mm) - (4 x angina index). Scores range from -25 to +15, defining the following categories: low risk (>5), intermediate risk (-11 to 4), and high risk (<-11). Patients classified as high or intermediate risk based on this score were referred to the cardiac catheterization laboratory for coronary angiography.

### Procedures

Coronary angiography was performed using Integris Allura Xper FD-20 systems (Philips Medical Systems, The Netherlands) and Artis Zee systems (Siemens Medical Systems, Germany), along with their respective quantification software for quantitative coronary angiography. This method is based on the principles of automatic arterial contour detection and image calibration. Patients were monitored in the hemodynamic care unit following the procedure and were discharged four hours later, provided no complications occurred. Follow-up was conducted over a three-year period through scheduled outpatient visits. In cases where patients were unable to attend, contact was maintained via telephone consultations or calls to family members. Data were obtained from the Angycor database, which operates with software designed for the automatic reporting and documentation of diagnostic and interventional procedures in the CIMEQ hemodynamics laboratory.

The sections referring to medical history, procedural reports, and follow-up consultations were obtained from the patient's medical record.

### Variables

The variables studied included age, sex, history of hypertension, diabetes *mellitus*, smoking, obesity, dyslipidemia, and chronic kidney disease. During follow-up, the incidence of adverse cardiovascular events was assessed, including cardiovascular mortality, acute coronary syndromes, hospitalization for heart failure, and stroke. Additional outcomes included the need for rehospitalization due to cardiovascular causes, the need for repeat coronary angiography, and event-free survival from major adverse cardiovascular events.

### Data Analysis

Frequency distribution tables and graphs were used to summarize the data, and statistical analysis was performed using SPSS (Statistical Package for the Social Sciences), version 21.0 for Windows. Summary measures included mean and standard deviation or median and interquartile range, depending on the distribution of each variable. Relative risk (RR) with corresponding confidence intervals was calculated. Survival analysis was performed using the nonparametric Kaplan-Meier method and the Log-Rank test (Mantel-Cox). A p-value < 0.05 was considered statistically significant in all analyses.

### Ethical considerations

This study complied with the principles outlined in the Declaration of Helsinki and the current legislation in Cuba governing research involving human subjects. Each participant received a detailed explanation of the study’s objectives, potential benefits, and risks, with clear emphasis on the voluntary nature of participation and the right to withdraw at any time. Written informed consent was obtained using a standardized consent form. The results will be used exclusively for scientific purposes.

## Results

A total of 110 patients were included. Prior to undergoing coronary angiography, they exhibited clinical characteristics such as a high prevalence of hypertension (58.2%), dyslipidemia (44.5%), and smoking (25.5%). Additionally, 24.5% of patients reported current smoking, and 21.8% had a family history of ischemic heart disease. The mean age was 64.5 ± 7.2 years, with a higher proportion of female patients (57%) (Table 1).

**Table 1:** Clinical and demographic characteristics of the study population.

Clinical and demographic Variables	n (%)
Age (Mean ± SD)	64.5 ± 7.2
Female sex	63 (57)
Hypertension	64 (58.2)
Diabetes *mellitus*	28 (25.5)
Dyslipidemia	49 (44.5)
Smoking	27 (24.5)
Obesity	19 (17.3)
Family history of IHD	24 (21.8)
Chronic kidney disease	5 (4.5)

SD: standard deviation. IHD: ischemic heart disease.

During a three-year follow-up period, adverse cardiovascular events were assessed, resulting in a cardiovascular mortality rate of 3.7%. Rehospitalizations occurred in 11% of cases, with heart failure identified as the leading cause, accounting for 10%. Additionally, 10% of patients required repeat angiographic procedures, mostly due to non-ST-segment elevation acute coronary syndrome, which affected 7.2% of patients. A relatively low incidence of ST-segment elevation acute coronary syndrome (2.7%) and stroke (0.9%) was observed (Table 2).

**Table 2:** Adverse cardiovascular events during three-year follow-up.

Adverse events	1 year	2 years	3 years	Total
	n (%)	n (%)	n (%)	n (%)
Rehospitalization	3 (2.7)	1 (0.9)	9 (8.2)	13 (11)
Repeat coronary angiography	3 (2.7)	1 (0.9)	7 (6.5)	11 (10)
STEMI	0 (0.0)	1 (0.9)	2 (1.9)	3 (2.7)
Non-STEMI	3 (2.7)	1 (0.9)	4 (3.7)	8 (7.2)
Stroke	0 (0.0)	1 (0.9)	0 (0.0)	1 (0.9)
Hospitalization for heart failure	2 (1.8)	1 (0.9)	7 (6.5)	11 (10)
Cardiovascular death	1 (0.9)	1 (0.9)	2 (1.9)	4 (3.7)

STEMI: ST-elevation myocardial infarction. NSTEMI: non-ST-elevation myocardial infarction.

**Figure 1:**
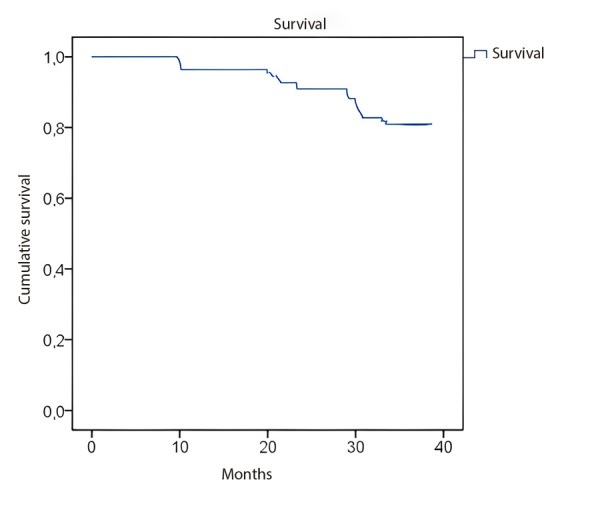
Event-free cardiac survival curve.

The three-year event-free cardiovascular survival rate reached 80% (Figure 1).

Diabetes mellitus (p = 0.040), family history of ischemic heart disease, and chronic kidney disease (p = 0.010), in that order, were the main cardiovascular risk factors associated with the occurrence of adverse cardiac events during follow-up (Table 3).

**Table 3:** Adverse cardiac events according to clinical and demographic variables.

Clinical and demographic variables	Adverse events		p value	RR (95% CI)
	Yes n (%)	No n (%)		
Female sex	13 (61.9)	50 (56.2)	0.630	0.7 (0.3 - 2.1)
Hypertension	14 (66.7)	50 (56.2)	0.380	1.5 (0.5 - 4.2)
Diabetes mellitus	9 (42.9)	19 (21.3)	0.040	2.7 (1.0 - 7.5)
Dyslipidemia	12 (57.1)	37 (41.6)	0.190	1.8 (0.7 - 4.9)
Smoking	8 (38.1)	19 (21.3)	0.110	2.3 (0.8 - 6.3)
Obesity	6 (28.6)	13 (14.6)	0.190	2.3 (0.8 - 7.1)
Family history of IHD	10 (47.6)	14 (15.7)	0.010	4.8 (1.7 - 13.6)
Chronic kidney disease	5 (23.8)	0 (0.0)	0.010	6.5(4.1 - 10.3)
Age (mean ± SD)	64.2 ± 8.3	64.6 ± 6.9	0.830	-

SD: standard deviation; RR: relative risk; CI: confidence interval; IHD: ischemic heart disease.

**Figure 2:**
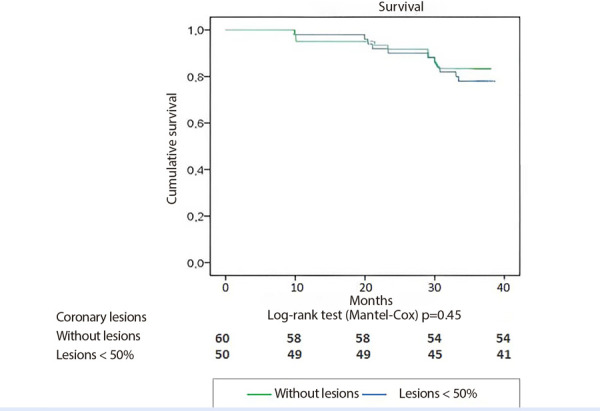
Event-free cardiac survival curve according to the presence of coronary lesions.

The event-free cardiovascular survival rate was similar between patients without coronary lesions and those with coronary lesions of less than 50% stenosis (Figure 2).

## Discussion

The absence of significant coronary lesions is a common finding in cardiac catheterization laboratories, with a reported incidence ranging from 35% to 50%, depending on the characteristics of the populations studied ^5^. A report by Patel *et al.* found lesions <50% in 37.6% of coronary angiograms performed in 397,954 patients with positive tests for myocardial ischemia ^10^. The mean age was 58 years, with 65% having hypertension, 56% dyslipidemia, 22% diabetes, and 20% reporting current smoking. These findings are consistent with those of the present study in terms of the distribution and frequency of coronary risk factors, with hypertension and dyslipidemia being the most prevalent, followed by diabetes mellitus and smoking. Although cardiovascular risk factors contribute to inflammation and oxidative stress, mechanisms that impair microvascular function, the direct link between these factors and the development of INOCA remains incompletely understood ^11^.

World Health Organization statistics on ischemic heart disease report a predominance in males between the sixth and seventh decades of life ^1^; however, the diagnosis of INOCA is more prevalent in younger women. In line with the reviewed literature, most of the study population in the present research was female ^3^^,^^5^^,^^6^. Data from the American College of Cardiology show that 51% of women and 32% of men undergoing coronary angiography do not have significant coronary lesions ^12^. Earlier reports considered patients with INOCA to be at low cardiovascular risk with a benign prognosis; however, more recent evidence reveals that this population faces a higher risk of cardiovascular events in the medium and long term, findings that are consistent with the results of the present study.

A subanalysis of the International Study of Comparative Health Effectiveness with Medical and Invasive Approaches (ISCHEMIA) revealed the absence of significant coronary lesions in 13% of patients, despite severe ischemia diagnosed through noninvasive testing. Among them, 50% reported recurrent chest pain and a marked decline in functional capacity and quality of life ^13^. A retrospective analysis of 11,223 patients with normal coronary arteries conducted in Denmark reported a high risk of rehospitalization and the need for repeat angiographic evaluations in this population ^14^.

The Women's Ischemia Syndrome Evaluation (WISE) study reported a 12.8% rate of cardiovascular mortality and acute myocardial infarction in women with INOCA over a 10-year follow-up period, identifying microvascular dysfunction as the primary underlying cause in most patients ^15^. Compared with women who had significant coronary lesions at baseline, those with INOCA had lower rates of myocardial infarction (MI) and cardiovascular death in the first year; however, the frequency of recurrent angina and repeat angiographic evaluations was significantly higher. In comparison with men, the rates of rehospitalization due to angina or acute coronary syndrome were four times higher in women with INOCA.

Reports indicate a higher prevalence of heart failure with preserved ejection fraction, as well as cerebrovascular events and microvascular dysfunction, in women. These conditions are associated with reduced quality of life, decreased functional capacity, and increased frequency of anginal episodes ^16^. The present study findings are consistent with these reports, as heart failure was the leading cause of rehospitalization. The Coronary Vasomotor Disorders International Study (COVADIS) has shown a progressive reduction in physical activity related to recurrent anginal episodes in patients with INOCA ^17^.

In this study, acute coronary syndromes were reported during follow-up, with a higher prevalence of non-ST-segment elevation acute coronary syndrome. The rupture or fissuring of an atherosclerotic plaque, regardless of whether it is hemodynamically significant, can trigger thrombosis or vasospasm, leading to reduced myocardial perfusion. Despite the variability in ischemic mechanisms not involving obstructive coronary lesions, multiple authors have reported increased morbidity and mortality associated with acute coronary syndromes ^18^^,^^19^.

The presence of nonobstructive lesions in all three coronary arteries carries an annual risk of acute myocardial infarction and death comparable to that of patients with significant single-vessel CAD ^18^. Consistent with findings from other authors, hypertension, diabetes mellitus, and smoking are associated with higher mortality ^18^. Sex, dyslipidemia, and a family history of CAD do not appear to be linked to mortality in patients with INOCA, according to several reports, but are associated with reduced quality of life due to diminished functional capacity and increased frequency of anginal episodes ^20^.

A limitation of the present study is the unavailability of advanced diagnostic tools, such as optical coherence tomography, intravascular ultrasound, coronary physiology testing, and cardiovascular magnetic resonance imaging, which hinders precise identification of the underlying mechanism of myocardial ischemia. Another notable limitation is that the results were derived from a single hospital-based institution, which may limit the generalizability of the findings to other healthcare settings.

In conclusion, patients with myocardial ischemia in the absence of obstructive coronary artery lesions experienced adverse events during follow-up. These events were more frequent among individuals with diabetes mellitus, a family history of ischemic heart disease, and chronic kidney disease.
